# Target localization accuracy in a respiratory phantom using BrainLab ExacTrac and 4DCT imaging

**DOI:** 10.1120/jacmp.v12i2.3296

**Published:** 2011-03-08

**Authors:** Jason E. Matney, Brent C. Parker, Daniel W. Neck, Greg Henkelmann, Isaac I. Rosen

**Affiliations:** ^1^ Department of Physics and Astronomy Louisiana State University Baton Rouge LA 70803‐4001 USA; ^2^ Mary Bird Perkins Cancer Center Baton Rouge LA 70809 USA

**Keywords:** respiratory motion, 4DCT, ExacTrac gating, implanted markers

## Abstract

This study evaluated the accuracy of measuring the motion of an internal target using four‐dimensional computed tomography (4DCT) scanning and the BrainLAB ExacTrac X‐ray imaging system. Displacements of a metal coil implanted in a commercial respiratory phantom were measured in each system and compared to the known motion. A commercial respiratory motion phantom containing a metal coil as a surrogate target was used. Phantom longitudinal motions were sinusoidal with a 4.0 second period and amplitudes ranging from 5–25 mm. We acquired 4DCT and ExacTrac images of the coil at specified respiratory phases and recorded the coordinates of the coil ends. Coil displacement relative to the 0% phase (full‐inhale) position were computed for the ExacTrac and 4DCT imaging systems. Coil displacements were compared to known displacements based on the phantom's sinusoidal motion. Coil length distortion due to 4DCT phase binning was compared to the known physical length of the coil (31 mm). The maximum localization error for both coil endpoints for all motion settings was 3.5 mm for the 4DCT and 0.8 mm for the ExacTrac gating system. Coil length errors measured on the 4DCT were less than 0.8 mm at end inhale/exhale phases, but up to 8.3 mm at mid‐inhalation phases at the largest motion amplitude (25 mm). Due to the fast image acquisition time (100 ms), no coil distortion was observable in the ExacTrac system. 4DCT showed problems imaging the coil during mid‐respiratory phases of higher velocity (phases 20%–30% and 70%–80%) due to distortion caused by residual motion within the 4DCT phase bin. The ExacTrac imaging system was able to accurately localize the coil in the respiratory phantom over all phases of respiration. For our clinic, where end‐respiration phases from 4DCT may be used for treatment planning calculations, the ExacTrac system is used to measure internal target motion. With the ExacTrac system, planning target size and motion uncertainties are minimized, potentially reducing internal target volume margins in gated radiotherapy.

PACS number: 87.56.‐v

## I. INTRODUCTION

The goal of radiation therapy is to maximize the absorbed dose in a target volume while minimizing dose to normal, healthy tissue. Conformal radiation therapy uses imaging modalities, such as computed tomography (CT), to define target volumes and 3D treatment planning software to generate plans that conform the dose distribution as closely as possible to those volumes. This dose conformation reduces complications by limiting the dose delivered to normal tissues.^(^ ^1^ ^,^ ^2^ ^)^ With a relative reduction of dose to normal tissues comes the ability to escalate the dose delivered to the target to increase tumor control probability while maintaining acceptable levels of normal tissue complications.^(^ ^3^ ^,^ ^4^ ^)^

One challenge for radiation therapy is compensating for respiratory organ motion. Currently, nongated standard radiation therapy techniques treat a volume that encompasses the tumor at all potential positions within the respiratory cycle.^(^ ^5^ ^,^ ^6^ ^)^ While this technique can adequately irradiate the entire target volume, it also increases the dose to surrounding normal tissues. Additionally, respiratory motion potentially reduces the localization accuracy of therapy delivered to targets in the thorax and abdomen. Accurate adjustment of the radiation delivery to compensate for respiratory motion will permit a reduction in the planning target volume and, therefore, reduce doses to surrounding normal tissues.

Potential techniques for incorporating intrafractional respiratory motion into treatment planning/delivery include breath‐hold,^(7)^ respiration‐gating,^(^ ^8^ ^–^ ^10^ ^)^ and target‐tracking.^(11)^ Breath‐hold techniques actively or passively suspend the patient's respiration for short intervals and deliver treatment during these intervals. However, patients with lung tumors can have compromised breathing capacity, and performing stable, extended breath holds for treatment may not be feasible. Respiration gating techniques periodically turn on the treatment beam when the patient's breathing parameters fall within a predefined range (such as near full‐inhale or full‐exhale phase of respiration). Target‐tracking (four‐dimensional) techniques propose to actively track the target with the radiation beam as the target moves during the respiratory cycle. However, target‐tracking four‐dimensional techniques greatly increase the complexity of the treatment delivery. All of these techniques require some form of observation of the respiratory motion. Current measurement techniques^(12)^ for tracking respiration include infrared external marker tracking, spirometry, strain gauges, video visual tracking, and fluoroscopic tracking of implanted fiducial markers.

The motion of a fiducial marker implanted into or near a tumor gives more accurate information about the motion of the tumor compared to external tracking techniques.^(13)^ However, the process of percutaneous or endoscopic implantation of fiducial markers is an invasive procedure. Furthermore, continuous monitoring of an implanted marker by X‐rays could result in a higher nontreatment dose to the patient. External motion sensors are safer and noninvasive, but may not correlate as well to internal tumor motions.^(14)^

This project investigated the accuracy of motions measured with both a 4DCT system required for treatment planning image acquisition and with the BrainLAB ExacTrac positioning system used for patient treatment positioning. Accurate target localization in both systems is required before gated treatments can be clinically implemented. Specifically, we measured the accuracy of localizing a moving fiducial marker implanted in a respiratory phantom moving with a known sinusoidal pattern. While a sinusoidal pattern does not exactly mimic human respiratory motion, it provides a simple and reproducible motion pattern with which to evaluate the localization capabilities of the systems under evaluation. Once the performance of these systems has been verified under ideal conditions, more complex and realistic human respiratory motion patterns can be evaluated in future studies.

With the ability to accurately track an implanted fiducial, the ExacTrac system will be able to accurately measure the extent of target motion, potentially aiding in the design of patient‐specific treatment margins. Also, the ExacTrac gating system uses an implanted fiducial marker for aligning the patient before treatment. In order to proceed with clinical implementation of the ExacTrac gating system, we need to investigate the accuracy of the system in resolving an implantable marker.

## II. MATERIALS AND METHODS

### A. Respiratory motion phantom

**Figure 1:**
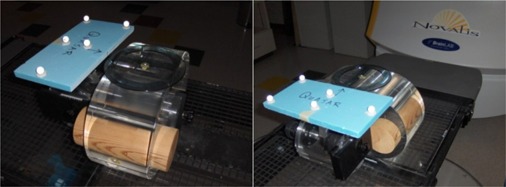
The Quasar respiratory motion phantom positioned on the Novalis treatment couch with five reflective BrainLAB bodymarkers used by the ExacTrac system to monitor the external chest wall motion.

The Quasar respiratory motion phantom (Modus Medical Devices, Ontario, Canada) is shown in Fig. 1 with a wooden (cedar) cylindrical insert designed to simulate lung tissue. Lung tissue has an average density of $0.2-0.5g\cdot {\text{cm}}^{-3}$. The phantom's wooden cylinder has a density near $0.3g\cdot {\text{cm}}^{-3}$. The average density of lung tissue also depends on the phase of respiration, and the manufacturer of the phantom selected the wood material as an intermediate density value. A brass coil (RadioMed Corporation, Tyngsboro, MA) with a diameter of 0.75 mm and a length of 31 mm was embedded into the outer surface of the wooden cylinder. The coil was positioned with its long axis in the superior–inferior direction, which was the direction of coil motion. This coil was used as a surrogate target for imaging to evaluate localization accuracy of the imaging systems. The rigid blue foam seen in Fig. 1 represents the patient chest wall, which moves in the anterior–posterior (AP) direction. The spheres attached to the platform are infrared reflecting markers, used for video monitoring.

The period and amplitude of the phantom's sinusoidal motion can be adjusted by the user. AAPM Report 91 recommends that respiratory gating should be considered when tumor respiratory motion exceeds 5 mm.^(12)^ Additionally, literature reports that, for most patients, a superior–inferior tumor movement over 2 cm is relatively uncommon.^(15)^ Thus, we evaluated phantom target motion amplitudes of 5, 10, 15, 20 and 25 mm motion in the longitudinal or superior–inferior direction of the phantom and 1 cm chest wall amplitude in the anterior– posterior direction.

**Figure 2:**
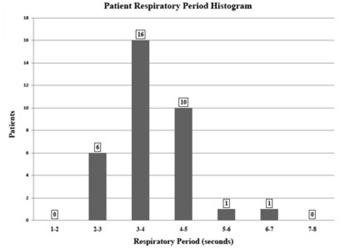
Respiratory period histogram from 34 patients scanned with clinical 4DCT protocol recorded by the Varian RPM system.

Existing clinical data were analyzed to select an appropriate respiratory period for the study. Of patients previously scanned with 4DCT clinical lung protocols, 34 patients had reproducible and sustained breathing traces. A histogram of the 34 patient respiratory periods is shown in Fig. 2. The majority (26 patients) were within the range of 3–5 seconds. The average for all 34 patients was 3.7±0.8 seconds. This correlates well with findings by Seppenwoolde et al.^(16)^ who reported an average respiratory period of 3.6±0.8 seconds. Thus, for this project, a phantom respiratory period of 4.0 seconds was used.

Additionally, the 4DCT system records the length of time the patient spent in exhale (~ 50% phase) and inhale (~ 0% phase). For the 34 patients, the average exhalation time was 2.1 seconds, compared to an average inhalation time of 1.5 seconds. This also agrees with respiratory observations reported by Seppenwoolde et al.^(16)^ who found that, on average, a patient spends more time in exhalation phases of respiration compared to inhalation phases.

### B. 4DCT acquisition and analysis

The phantom 4DCT data were acquired using cine CT scanning in conjunction with the Varian Real‐Time Positioning Management (RPM) System (Varian Oncology Systems, Palo Alto, CA). During scan acquisition, chest wall motion (based on the motion of an infrared reflecting marker box) is recorded by the RPM system. Once the scan is completed, the reconstructed CT images and the RPM chest wall motion data are sent to a GE Advantage (General Electric Company, Waukesha, WI) computer for sorting. The sorting program correlates the timestamp on each CT image to the RPM file to determine the relative chest wall amplitude and respiratory phase for each image. The images are then sorted into ten distinct phases ranging from full inhale (0%) to full exhale (50%) and back to inhale. The phase‐sorted images are combined to create a 3D CT data set for each phase.^(17)^ Pan et al.^(^ ^18^ ^,^ ^19^ ^)^ provide an in‐depth description of the process to create the phase‐sorted images formed during 4DCT.

To evaluate the effects of respiratory motion on the 4DCT images, the phantom was scanned with coil motion amplitudes of 5, 10, 15, 20, and 25 mm using our clinical 4DCT protocol (2.5 mm slice thickness, 120 kV, 440 mA, 6.0 second cine duration). The long axis of the coil and coil motion were perpendicular to the axial CT scan planes. For each scan, a 50 cm field of view was used, with 512×512 pixel resolution. Therefore, the voxel size in each slice was 0.98×0.98×2.5 mm^3^. All 4DCT datasets were imported into the Pinnacle treatment planning system (Philips Medical Systems, Andover, MA) for analysis. Utilizing a bone‐optimized viewing window, the superior and inferior ends of the implanted coil were identified on each phase. Each end was defined as a point‐of‐interest (POI) in Pinnacle. The CT coordinates (AP, LAT, and SI) of the POIs were used to calculate the coil length in each phase and the three dimensional displacements of the ends relative to the 0% phase. The measured coil endpoints were compared to the known physical coil length and expected coil displacement to determine the error in imaging in each phase. The expected coil displacement was calculated from the period and amplitude of the phantom's sinusoidal motion. The expected motion was verified by measurement.

### C. ExacTrac image acquisition and analysis

The ExacTrac gating system uses a combination of infrared, optical and X‐ray tracking to align the patient and gate the treatment beam. The system is installed on a Novalis (BrainLAB, Munich, Germany) treatment unit. Initially, the system establishes the relationship between the infrared reflecting external markers and the implanted marker(s). It has been shown that marker migration once implanted into a patient is minimal.^(20)^ Then, the system monitors external markers and only allows radiation delivery when they are within a predetermined window of respiratory amplitude. The camera system consists of two stereoscopic infrared cameras and a video camera for visual monitoring, mounted on a rail above the foot of the treatment couch. The breathing signal tracked by the ExacTrac system is the time‐dependent vertical (anterior– posterior) movement of markers attached to the patient (or phantom in this study) as seen by the infrared cameras. Yan et al.^(21)^ provide a description of how the ExacTrac system resolves the three‐dimensional position of the markers from the two‐dimensional camera information.

**Figure 3:**
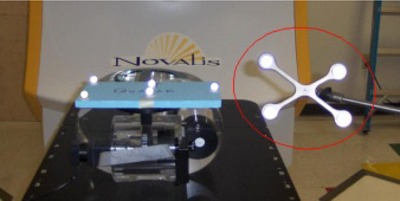
The ExacTrac Reference Star (circled in red) is shown mounted next to the Quasar phantom on the Novalis treatment couch.

To eliminate the influence of couch movement on the observed breathing trace, a stationary reference object is also monitored by the cameras. BrainLAB has developed the “Reference Star”, shown in Fig. 3, which consists of four infrared‐reflecting pads in a known configuration held stationary by an arm mounted to the side of the treatment couch. The ExacTrac system observes the moving patient‐mounted markers, shown on the Quasar phantom in Fig. 1, and the reflecting pads of the Reference Star. The breathing motion is calculated from the motion of the external patient markers relative to the “Reference Star” stationary markers.

ExacTrac uses a predictive breathing algorithm to acquire stereoscopic X‐ray images for 3D localization of the target at an expected position. This algorithm uses the motion of the infrared reflecting markers to predict when the moving target will be in a particular location. The algorithm times the X‐ray acquisition so that the moving target will be at a predicted location halfway through the X‐ray exposure. For all images taken with the ExacTrac system, the X‐ray exposure time is 100 ms, so the predictive algorithm begins X‐ray acquisition 50 ms before the moving target is at the desired location, ending 50 ms after the target is at the desired location. Unlike the 4DCT system which images in terms of phase within the respiratory cycle, the ExacTrac predictive algorithm operates in terms of external marker motion amplitude. It cues off respiratory motion from full inhale (0% Phase, 100% Amplitude) to full exhale (50% Phase, 0% Amplitude). Thus, the ExacTrac imaging system can only produce images which would correspond to 4DCT phase images from 0% to 50% phase. Only the motion between these phases can be compared in both ExacTrac and 4DCT.

Based on the sinusoidal motion of the phantom, the phase of the 4DCT images can be correlated with the amplitudes of ExacTrac images. Calculation shows that from inhale to exhale, relative amplitude levels of 100, 90.5, 65.5, 34.5, 9.5, and 0 percent correspond, respectively, to phases of 0, 10, 20, 30, 40 and 50 percent for a sinusoidal curve. These respiratory amplitude levels were used to acquire ExacTrac X‐ray images. Therefore, coil displacements and distortions measured with the ExacTrac system were directly compared to measurements from the corresponding 4DCT phase defined images.

**Figure 4:**
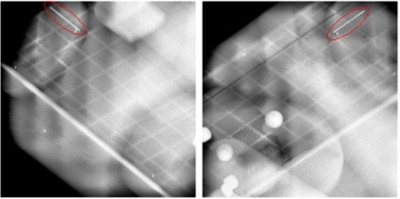
A pair of orthogonal X‐ray images taken with the ExacTrac system. The implanted coil (circled in red) is seen at the top left in the left image and at the top right in the right image.

The positions of the coil endpoints were identified on the ExacTrac X‐ray images. From this information, the ExacTrac system calculated the three‐dimensional positions of the coil endpoints for each respiratory amplitude level. This process was repeated for each of the motion amplitudes (5–25 mm) used in this study. The position of the coil at the time of image acquisition was compared to the 100% amplitude (0% phase) position to determine its 3D displacement. The measured displacement was then compared to the known displacement of the coil. Figure 4 shows a sample set of X‐ray images taken by the ExacTrac system of the Quasar respiratory phantom with the implanted coil.

## III. RESULTS

Table 1 shows the errors in coil length measured from 4DCT images. A similar table does not exist for the ExacTrac system because the X‐ray images are near instantaneous (100 ms) exposures of the coil, which have minimal motion blurring. The scanner resolved the true length of the coil to within 0.8 mm at end‐inhalation and end‐exhalation respiratory phases (0 and 50% phase, respectively) for all motion amplitudes. At these phase locations, measured coil length is limited by the resolution of the CT slice thickness (2.5 mm). When the coil was moving faster at mid‐inhalation/exhalation, coil length errors up to 5.3 mm were observed. As expected, the error in measured coil length generally increased with increasing motion amplitude because of greater residual motion within the 4DCT image binning window. The period remained the same for all amplitudes, so the coil velocity increased with increasing amplitude. There is a sharp increase in measured coil length error between the 20 mm and 25 mm amplitudes for the 20% and 30% phases. There were no obvious causes observed in the performance of this study. A smaller CT slice thickness should reduce this error, but there may be additional causes. Additional work will be required to determine the cause of this large increase in the measured coil length.

**Table 1:** Errors in coil length measured in mm from 4DCT for all phases and motion amplitudes.

*Phase*	*5 mm*	*10 mm*	*15 mm*	*20 mm*	*25 mm*
0%	0.0	0.5	0.7	0.7	0.4
10%	0.7	0.1	1.1	1.1	0.6
20%	0.6	1.1	1.6	1.2	8.1
30%	0.6	1.0	0.5	1.2	8.3
40%	0.4	1.0	1.4	0.9	1.5
50%	0.2	0.3	0.8	0.1	0.7
60%	0.0	0.1	1.4	0.8	0.6
70%	0.3	1.1	1.9	3.4	2.9
80%	0.3	1.6	1.3	1.5	3.4
90%	0.5	0.8	0.0	2.2	1.3

The measured errors in coil displacement for both 4DCT and ExacTrac at all amplitudes are shown in Table 2. The full‐exhale phase was selected as the reference position. The measured positions of the superior and inferior endpoints were used to determine the midpoint of the coil in 4DCT, and this value differed by up to 3.5 mm from the expected position. The maximum error occurred at the largest amplitude (25 mm) and at the highest velocity (mid‐respiration).

**Table 2:** Error in positions of coil midpoints (a‐e) relative to full‐exhale (0% amplitude, 50% phase). Measurements were made on the 4DCT data and ExacTrac trials. Table values are expressed in mm.

a. Measured Position Error (5 mm motion)			
*ExacTrac*	*CT Phase*	*4DCT*	*ET*
0%	50%	‐	‐
9.5%	40%	0.3	−0.1
34.5%	30%	−0.4	0.2
65.5%	20%	0.7	0.2
90.5%	10%	0.1	0.2
100%	0%	0.5	0.1
b. Measured Position Error (10 mm motion)			
*ExacTrac*	*CT Phase*	*4DCT*	*ET*
0%	50%	‐	‐
9.5%	40%	−0.3	−0.2
34.5%	30%	−1.2	0.2
65.5%	20%	−0.2	0.3
90.5%	10%	−0.1	0.2
100%	0%	−0.1	0.2
c. Measured Position Error (15 mm motion)			
*ExacTrac*	*CT Phase*	*4DCT*	*ET*
0%	50%	‐	‐
9.5%	40%	−0.7	0.3
34.5%	30%	−2.9	0.7
65.5%	20%	−3.5	0.8
90.5%	10%	−1.2	0.4
100%	0%	−1.2	0.5
d. Measured Position Error (20 mm motion)			
*ExacTrac*	*CT Phase*	*4DCT*	*ET*
0%	50%	‐	‐
9.5%	40%	−1.3	−0.2
34.5%	30%	0.1	0.5
65.5%	20%	−1.8	0.4
90.5%	10%	−0.9	−0.1
100%	0%	−1.4	0.1
e. Measured Position Error (25 mm motion)			
*ExacTrac*	*CT Phase*	*4DCT*	*ET*
0.%	50%	‐	‐
9.5%	40%	0.0	0.1
34.5%	30%	−0.5	0.5
65.5%	20%	−1.8	0.7
90.5%	10%	−0.2	0.1
100%	0%	−1	−0.2

The ExacTrac system does not give the user the location of the coil endpoints, but a “center of mass” of the coil points. Two ExacTrac trials were averaged to provide displacement data to contrast with the coil displacement studies from the 4DCT. All ExacTrac measurements were within 0.8 mm for all phases of respiration and 5 amplitudes.

## IV. DISCUSSION

For tracking the position of an implanted coil during sinusoidal motion, the ExacTrac system performed better than 4DCT imaging. The ExacTrac performs better because the X‐ray exposure is a near‐instantaneous image of the coil, instead of a reconstruction using a phase bin with finite temporal width. The ExacTrac system localized the coil to within a millimeter (0.8 mm) of the expected position, while 4DCT registered errors up to 3.5 mm. 4DCT showed problems resolving the coil length during large respiratory‐induced velocities with errors up to 8.3 mm of actual coil length, but accurately resolved the coil length to within 1 mm of actual coil length at end expiration/inhalation.

It is expected that there may be some differences in the results using a phantom motion that more closely mimics human respiratory motion. Since human respiratory motion is not sinusoidal but spends more time in the exhalation phases, coil velocity would be smaller during this time. This should result in less residual motion within the phase bins during 4DCT and potentially a smaller localization error for 4DCT. However, since the breathing period used in this study was based on human data, a longer exhale phase than the sinusoidal motion used here would result in a shorter inhale phase than simulated with the sinusoidal motion. This would result in a higher coil velocity during inhale than was used in this study, potentially leading to even larger errors in localization during the inhale phase. Another study using more realistic human breathing patterns is being planned. For 4DCT evaluation, a more human respiratory motion profile is not expected to change the results of the ExacTrac study since its image acquisition time is already so short (~ 100 ms).

As expected, the 4DCT gives a more accurate image of the coil at end‐exhalation/inspiration (compared to mid‐respiration) where residual motion within the binning window is the least. This indicates that the 4DCT produces the most accurate image of the moving phantom at phases corresponding to end‐respiration. Since exhale is generally considered to be a more stable, longer‐lasting phase of respiration and results in minimal anatomic distortion, this work recommends using the full‐exhale respiratory phase for our clinic's respiratory‐gated treatment planning.

Throughout all phases of the breathing cycle, 4DCT target localization is limited by the slice thickness used for image acquisition. The use of a smaller slice thickness should improve the localization accuracy to some extent. However, some of the measured 4DCT localization errors were significantly larger than the uncertainty of half of the slice thickness. This indicates that residual motion within the phase bins is the primary cause of these errors as they occurred during phases where coil velocity was highest. Smaller CT slices would reduce the spatial resolution in the slice thickness directions, but would not reduce the residual motion within the imaging phase bins, as this is limited by temporal resolution and not spatial resolution.

## V. CONCLUSIONS

To accurately determine the direction and extent of internal respiratory motion after coil implantation, this work recommends using the ExacTrac system. For 4DCT acquisition, we recommend that our physicians use a 1.25 mm slice thickness for 4DCT imaging to reduce localization errors as much as possible. For a patient with well‐behaved chest wall motion (i.e., no coughing, gasping, or sudden motions), the ExacTrac system should be able to localize an implanted coil to within 1 mm. The extent of tumor motion could be directly measured for use in treatment planning and for the selection of gating parameters. The submillimeter agreement of techniques outlined in this work may allow users to better define margins in radiotherapy.

## ACKNOWLEDGMENTS

The authors acknowledge the Louisiana State University Board of Regents for their financial support for this project. This work was supported in part by a research agreement with BrainLAB, Inc.
